# The role of *Sophora alopecuroides* alkaloids in colon health of lambs fed high-concentrate diets for extended periods: impact on barrier function, antioxidation, and microflora

**DOI:** 10.3389/fvets.2025.1698892

**Published:** 2025-12-10

**Authors:** Shufang Li, Boyang Li, Henan Lu, Jianxin Zhao, Aiwu Gao, Yawen An, Jinli Yang, Hairong Wang

**Affiliations:** 1 Animal Nutrition and Feed Science, Inner Mongolia Agricultural University, Hohhot, China; 2 LFood Science, Inner Mongolia Agricultural University, Hohhot, China; 3 Veterinary Research Institute, Inner Mongolia Academy of Agricultural & Animal Husbandry Sciences, Hohhot, China

**Keywords:** microflora, high-concentrate diets, colonic epithelium, *Sophora alopecuroides* alkaloids, lambs

## Abstract

**Introduction:**

Long-term feeding of a high-concentrate diet can induce subacute ruminal acidosis (SARA) and hindgut acidosis in ruminants. However, at present, most studies focus on reducing rumen injury by adjusting the feed formula, adding buffers, probiotics, or enzyme preparations, and few studies pay attention to hindgut health. *Sophora alopecuroides* alkaloids have extensive anti-inflammatory and antioxidant effects. The purpose of this experiment was to study the effects of adding total alkaloids of *Sophora alopecuroides* (TASA) to a high-concentrate diet on colon barrier function, antioxidation, and the microbial flora of lambs.

**Methods:**

18 Dumont lambs (26.37 ± 2.29 kg) were divided into three diet groups: medium-concentrate diet (MC, concentrate ratio 50:50), high-concentrate diet (HC, concentrate ratio 70:30), and HC diet supplemented with 121 mg/kg TASA (HCT). At the end of the experimental period, colon contents and colon epithelium were collected. These samples were used to evaluate the colon barrier, antioxidant capacity, intestinal morphology, microbial composition and short-chain fatty acid (SCFA) concentration.

**Results:**

The results revealed that adding TASA to the HC diet increased claudin-1 protein expression (*p* < 0.01), decreased the MDA concentration, and increased Glutathione peroxidase (GSH-Px), Superoxide dismutase (SOD), and Total antioxidant capacity (T-AOC) activity in the colonic epithelium (*p* < 0.05). The concentration of propionate and lactate in colon contents in HC group increased significantly, while the pH decreased significantly (*p* < 0.05). The concentration of acetate, propionate and lactate in HCT group was significantly lower than that in HC group, the concentration of butyrate in HCT group was the highest (*p* < 0.05). Furthermore, there was a significant increase in Bacteroidetes and a decrease in Firmicutes in the HCT group (*p* < 0.01). Compared with the HC group, there was a notable increase in the butyrate-producing genera *Faecalibacterium*, *Roseburia*, *Lachnospiraceae_NK4A136_group*, and *Butyrivibrio* in the HCT group (*p* < 0.05 or *p* < 0.01). Additionally, the abundances of *Prevotellaceae_UCG-003* in the MC and HCT groups were significantly greater (*p* < 0.05 or *p* < 0.01).

**Conclusion:**

In conclusion, supplementing the HC diet with TASA enhances colonic barrier and antioxidant functions, and alleviates HC diet-induced colonic damage by modulating the structure and abundance of the colonic microbiota.

## 1 Introduction

To maximize economic benefits, livestock farms typically adopt HC diet strategies. However, feeding of HC diets for a prolonged duration leads to subacute ruminal acidosis (SARA), impairing barrier function and inducing ruminal or systemic inflammation (1). This condition adversely affects the health of ruminants, exacerbating animal welfare issues and environmental challenges. Additionally, the abnormal ruminal function caused by HC diets leads to an increased influx of fermentable carbohydrates into the small intestine and hindgut (2). The colon, which is composed of a single layer of columnar epithelium and lacks the salivary bicarbonate buffering capacity found in the rumen, is more susceptible to metabolic disturbances caused by the HC diet (3). Numerous studies have confirmed that when ruminants are fed HC diets, there is a decrease in colonic pH, an increase in lactic acid and volatile fatty acid (VFA) concentrations, and a disruption of the microbial balance. This disruption is marked by a reduction in beneficial bacteria and an increase in pathogenic bacteria (4, 5). Studies have shown that feeding HC diets increases the lipopolysaccharide (LPS) concentration, decreases tight junction protein expression in the colonic epithelium, and promotes proinflammatory cytokine secretion (3). Furthermore, oxidative stress is also a key factor in colonic damage induced by HC diets. Studies have shown that under HC diet conditions, colonic epithelial damage and oxidative stress are related to increased apoptosis. HC diets increase MDA levels in the colon, decrease GSH-Px and SOD activities, and downregulate bcl2 while upregulating bax, caspase-3, and caspase-8 mRNA expression (6). However, in light of the comprehensive efforts to prevent resistance at the feed end and the frequent occurrence of nutritional metabolic diseases in ruminants due to HC diets in China, addressing the issue of resistance substitution and maintaining the health of animals are key challenges for the feed industry and nutritional regulation.

*Sophora alopecuroides* (SA) is a perennial medicinal plant that is traditionally used to treat gastrointestinal diseases, including dysentery and enteritis, in which alkaloids are the primary chemical components. SA alkaloids regulate microbiota composition (7, 8), inflammatory reactions (7, 9), oxidative stress (10), and metabolism (11) while promoting intestinal stem cell differentiation (12) to maintain normal intestinal barrier function. Additionally, our previous research found that SA and its alkaloids can improve the growth performance of lambs when fed HC diets, regulate the structure of the rumen flora, and improve the immune and antioxidant functions of the body (13, 14). At the same time, it performs the function of maintaining the gastrointestinal barrier and protecting the liver (15, 16). Therefore, this study examined the effects of adding total alkaloids of *Sophora alopecuroides* (TASA) to HC diets on colonic barrier function, antioxidants, and microflora in lambs. The findings provide new strategies for mitigating colonic damage induced by HC diets.

## 2 Materials and methods

### 2.1 Experimental design

A total of 18 Dumont lambs (weight 26.37 ± 2.29 kg) were randomly divided into three dietary treatment groups: a medium-concentrate diet (MC; concentrate/roughage 50:50), a high-concentrate diet (HC; concentrate/roughage 70:30), and an HC diet supplemented with 121 mg/kg TASA on a dry matter basis (HCT). The TASA contained an alkaloid content of 95.1%, including 46.7% sophoridine. Table 1 displays the experimental diet composition and nutritional level. The pretest period was 15 days, and the full test period was 60 days. The barn was thoroughly disinfected, and the experimental sheep were marked prior to the trial. The remaining feed in the trough was weighed at 08:00 every day from the previous day. The lambs were fed twice daily at 09:00 and 18:00 to ensure free access to food and water, with the daily surplus maintained at over 15% of the total feed intake to ensure adequate supply.

**Table 1:** Composition and nutrient levels of the experimental diets (DM basis, %).

Items	Diets	
	MC	HC
Ingredients (% of DM)		
Mixed hay	50.00	30.00
Corn	36.10	52.00
Soybean meal	12.00	15.20
Limestone	0.60	1.00
CaHPO_4_	0.10	0.10
Nacl	0.50	0.50
Premix^1^	0.20	0.20
NaHCO_3_	0.50	1.00
Total	100.00	100.00
Nutrient levels (%)		
Crude protein	14.17	14.45
Neutral detergent fiber	33.18	25.03
Calcium	0.77	0.71
Phosphorus	0.38	0.37
Acid detergent fiber	22.36	15.37
Metabolizable energy (MJ/kg)^2^	9.53	10.20

^1^Provided per kg of premix: Fe 25.0 mg; I 0.90 mg; Zn 35.0 mg; Cu 9.0 mg; Co 0.1 mg; Se 0.25 mg; Mn 19.5 mg; nicotinic acid 60 mg; vitamin E 15 U; vitamin A 3000 U; vitamin D_3_ 1,000 U. ^2^Metabolizable energy is a calculated value, and the rest of the nutritional indicators are measured values.

### 2.2 Sample collection

After the rearing period, the lambs were slaughtered in the slaughter room at the practice base of Inner Mongolia Agricultural University. First, a 1 ~ 2 cm long colon segment was cut and fixed in 4% paraformaldehyde for paraffin sectioning. Then, sterile spoons were used to scrape the colonic contents, which were frozen in liquid nitrogen for later analysis of VFAs and microbial sequencing. Finally, the colonic tissue was rinsed with 0.9% saline, and the colonic epithelium was gently scraped and placed into cryovials for storage at −80 °C.

### 2.3 Histological analysis of colon tissue

Colon tissue was paraffin-embedded and stained with H&E using standard techniques. Morphology was examined using an upright Nikon microscope (Nikon, Japan).

### 2.4 Western blot analysis

Total proteins were extracted from the colon tissues by grinding with liquid nitrogen and lysing with the RIPA lysis solution on ice for 30 min. The total proteins were quantified using the BCA method, and then the extracted proteins were sequentially subjected to 12.5% SDS–PAGE, followed by incubation with primary antibodies [ZO-1 (Proteintech, China), occludin (China), claudin-1 (Beyotime Biotechnology, China), and *β*-actin (Beyotime Biotechnology, China)]. After washing, they were incubated with secondary antibodies (goat anti-rabbit IgG polymer, LI–COR, United States) diluted 1:10,000, followed by washing with PBST. The samples were washed with PBST, and the target protein expression was scanned and analyzed using the LI-COR Odyssey infrared fluorescence imaging system (LI-COR, United States). *β*-actin was used as the reference protein to normalize the results of the target proteins, and the gray value ratio of the target protein to the reference protein was used as the relative expression of the target protein.

### 2.5 Measurement of antioxidant indices in the colon

The total protein concentration and the levels of MDA, CAT, GSH-Px, T-SOD, and SOD were determined according to the instructions provided (Nanjing Jiancheng Bioengineering Institute).

### 2.6 Measurement of the VFA concentration

According to the gas chromatography method described by Tao et al. (17), VFAs in the colon were determined as follows: Weigh 2.0 g of the colonic contents, add 2 mL of distilled water, thaw completely at 4 °C, and mix by vortexing. Then, pipette 1.5 mL of the supernatant, add 200 μL of 2-EB, vortex to mix, and centrifuge at 10,000 × g at 4 °C for 20 min. Collect the supernatant and filter it into a sample bottle with a 0.22 μM filter membrane. Finally, VFAs in the colonic contents were determined using a gas chromatograph (GC-7890B, Agilent Technologies).

### 2.7 Colonic microbial diversity analysis

Total DNA was extracted from the colonic contents using a Magen kit (Magen, China). After confirming its concentration and purity with an ND-2000 (NanoDrop Technologies), sequencing was performed on the Illumina MiSeq platform (Illumina, CA, United States). The sequence obtained after sequencing was filtered by fastp, and the DADA2 algorithm was used to denoise the valid sequences to obtain amplicon sequence variants (ASVs). We employed QIIME 2 and R 4.2.0 software to execute different analyses between alpha diversity index groups. The Kruskal–Wallis test was used to analyze differences in bacterial phylum and genus levels among the MC, HC, and HCT groups. PCoA with the Bray–Curtis distance algorithm was used to assess the microbial community structure similarity among the MC, HC, and HCT groups, and the PERMANOVA non-parametric test was used to assess the significance of differences in microbial community structure among the three groups. Linear discriminant analysis (LDA) was conducted to identify genera that were significantly abundant across the MC, HC, and HCT groups (LDA score > 4, *p <* 0.05).

### 2.8 Statistical analyses

One-way ANOVA using SPSS 23 (IBM Corporation, NY, United States) was performed to analyze colonic inflammatory factor levels, tight junction protein expression, antioxidant enzyme activities, and fermentation parameters. Bar charts were created with GraphPad Prism 8.0.1 (version 8.0.1, GraphPad, United States). Significance was set at a *p*-value of < 0.05, with a *p*-value of < 0.01 indicating high significance.

## 3 Results

### 3.1 Effect of TASA on the colonic epithelial morphology of lambs

**Figure 1:**
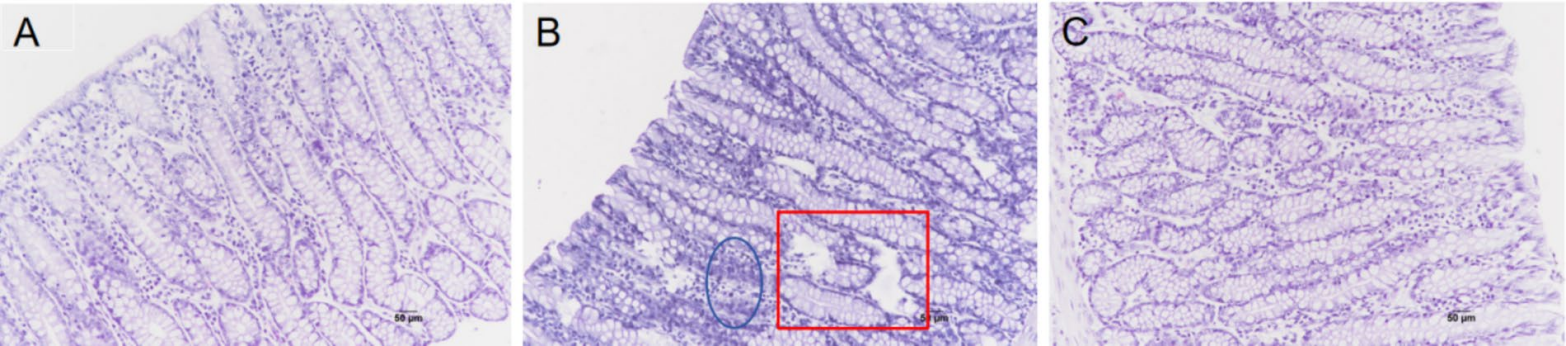
Effect of TASA on the morphology of the colonic epithelium in the lambs (HE × 200). **(A)** MC group. **(B)** HC group; squares represent epithelial cavities, and ellipses represent inflammatory cells such as eosinophils. **(C)** HCT group.

As depicted in Figure 1, compared to the MC group, the colonic epithelium in the HC group displayed cavities and inflammatory cell infiltration, whereas the addition of TASA to the HC group resulted in reduced inflammatory cell infiltration.

### 3.2 Effect of TASA on the relative expression level of tight junction proteins in the lamb colonic epithelium

**Figure 2:**
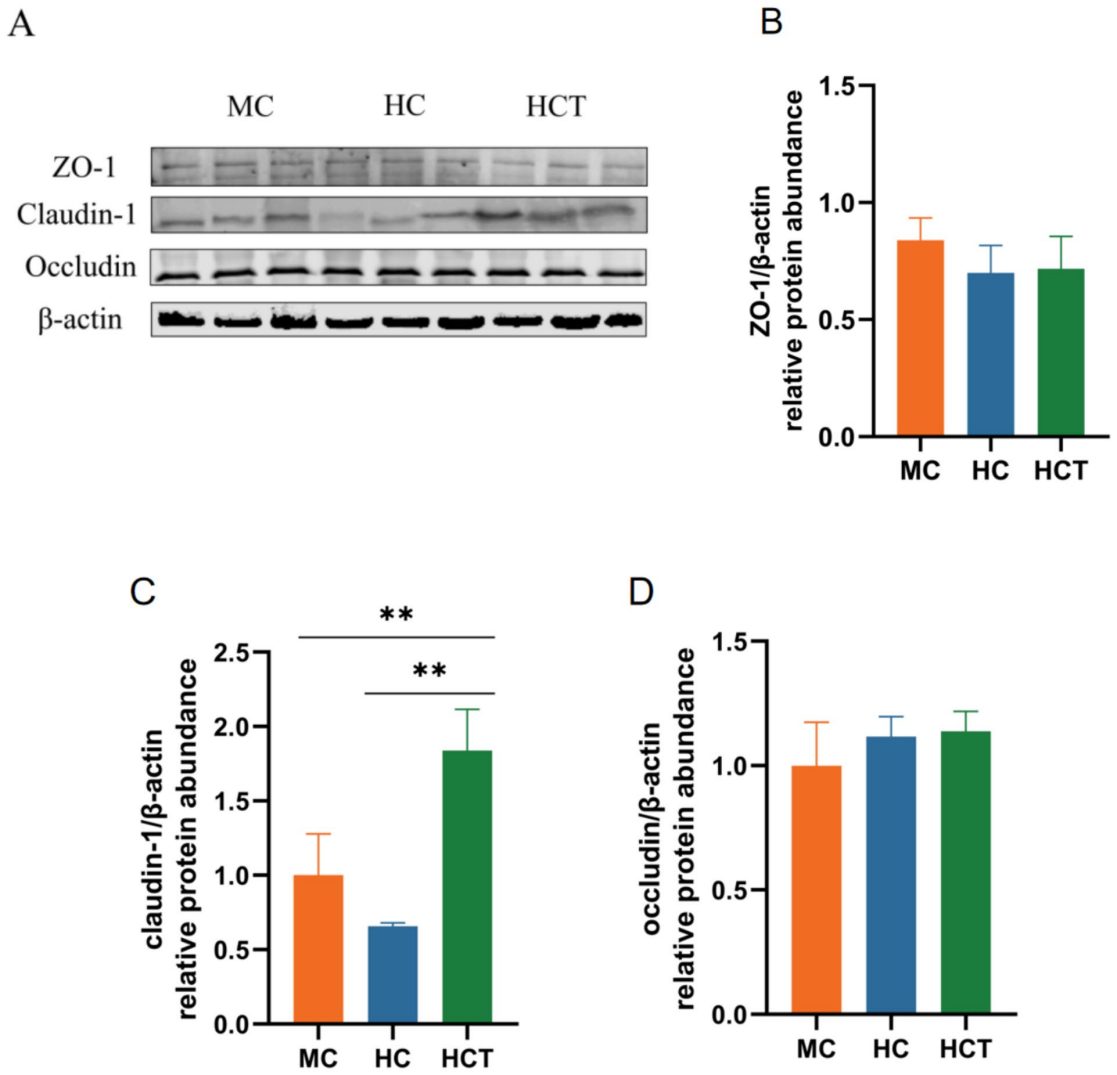
Effect of TASA on tight junction protein levels in the colonic epithelium of the lambs. **(A)** Western blot analysis. Relative protein abundance of **(B)** ZO-1, **(C)** claudin-1, and **(D)** occludin. * *p* < 0.05, ** *p* < 0.01. MC = (concentrate: roughage 50:50) diet; HC = (concentrate: roughage 70:30) diet; HCT = HC supplemented with 0.121 g/kg TASA.

Western blot images showed the protein expressions of ZO-1, Claudin-1, Occludin and β-actin inthree groups (Figure 2A). The ZO-1 and occludin protein levels in the lamb colonic epithelium were consistent across all groups (*p* < 0.05, Figures 2B,D). Claudin-1 protein expression in the colonic epithelium was significantly greater in the HCT group than in the MC and HC groups (*p* < 0.05, Figure 2C).

### 3.3 Effect of TASA on antioxidant indices in the lamb colon

**Figure 3:**
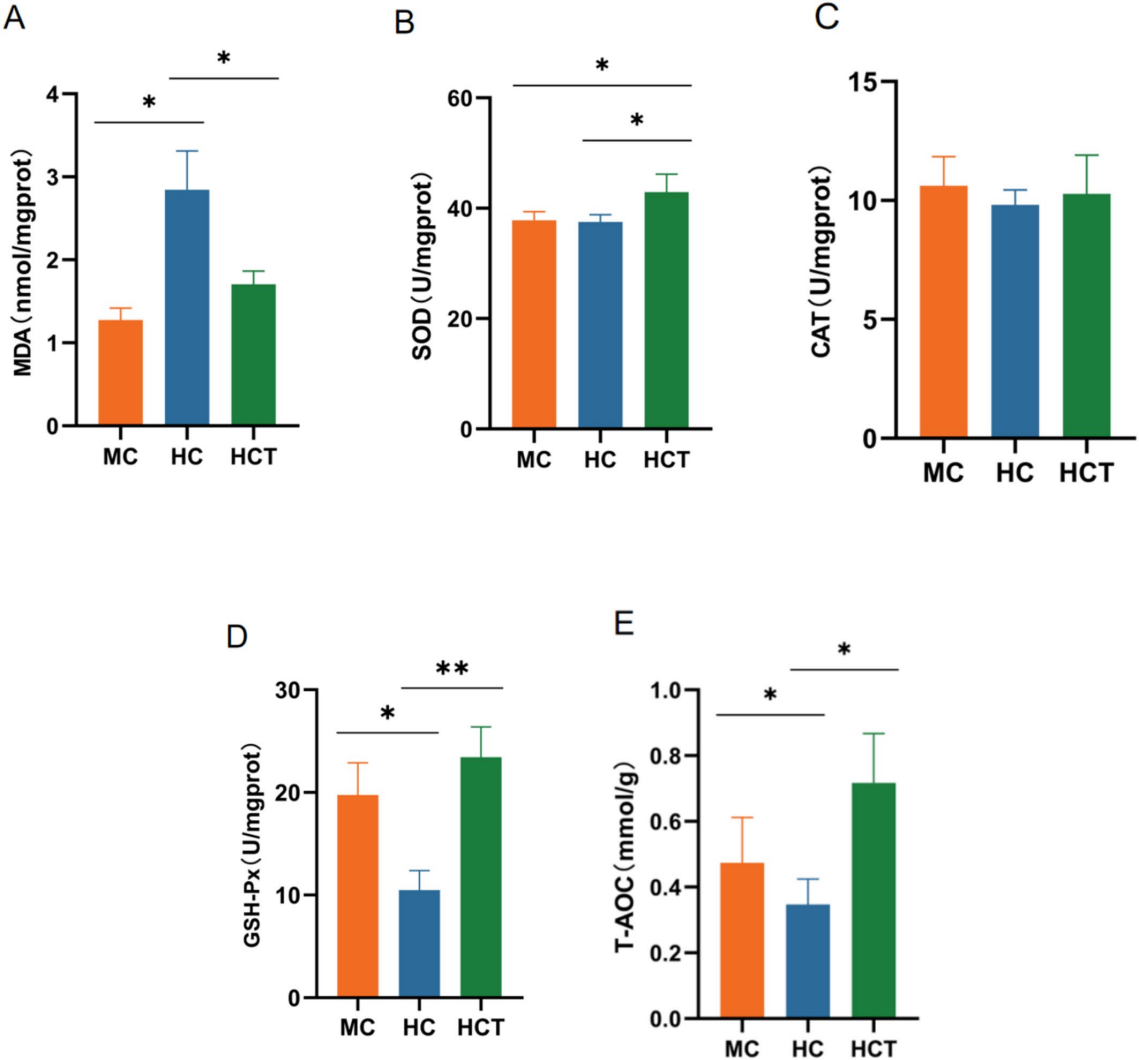
Effect of TASA on antioxidant function in the lamb colon. The concentrations of colonic epithelial **(A)** MDA, **(B)** SOD, **(C)** CAT, **(D)** GSH-Px, and **(E)** T-AOC. * *p* < 0.05, ** *p* < 0.01. MC = (concentrate: roughage 50:50) diet; HC = (concentrate: roughage 70:30) diet; HCT = HC supplemented with 0.121 g/kg TASA.

The MDA concentration in the colonic epithelium of the lambs in the MC and HCT groups was considerably lower compared to the HC group (*p* < 0.05, Figure 3A). The analysis did not reveal any significant differences in CAT activity across the groups (*p* > 0.05, Figure 3C). Compared to the MC group, the HC group showed significantly lower GSH-Px activity and T-AOC levels (*p* < 0.05), whereas the HCT group demonstrated significantly higher levels compared to the HC group (*p* < 0.05 or *p* < 0.01, Figures 3D,E). SOD activity was significantly higher in the HCT group than in the MC and HC groups (*p* < 0.05, Figure 3B).

### 3.4 Effect of HCT on the VFA content in the colons of lambs

As demonstrated in Table 2, the HC group showed a significantly lower pH value and higher propionate concentration than the MC and HCT groups (*p* < 0.05). In the HCT group, acetate levels were significantly lower (*p* < 0.05), butyrate levels were significantly higher than those in the MC group (*p* < 0.05), and lactate levels were significantly lower than those in the HC group (*p* < 0.05).

**Table 2:** Effect of TASA on the parameters of colon fermentation in the lambs.

Items	Groups			SEM	*P*-value
	MC	HC	HCT		
pH	6.69^a^	6.36^b^	6.66^a^	0.58	0.007
Acetate (mmol/g)	28.13^a^	26.45^a^	16.26^b^	2.27	0.050
Propionate (mmol/g)	5.53^b^	7.50^a^	4.91^b^	0.35	0.048
Butyrate (mmol/g)	1.90^ab^	1.63^b^	3.03^a^	0.27	0.042
Isobutyrate (mmol/g)	0.28	0.39	0.40	0.06	0.719
Valerate (mmol/g)	0.19	0.20	0.20	0.02	0.924
Isovalerate (mmol/g)	0.29	0.37	0.38	0.06	0.866
Lactate (mmol/g)	0.78^ab^	1.00^a^	0.56^b^	0.07	0.014

Data were expressed as mean ± SEM. In the same row, values with different letter superscripts mean a significant difference (*p* < 0.05). MC = (concentrate: roughage 50:50) diet; HC = (concentrate: roughage 70:30) diet; HCT = HC supplemented with 0.121 g/kg TASA.

### 3.5 Richness, diversity estimates, and bacterial composition in the colons of lambs

**Figure 4:**
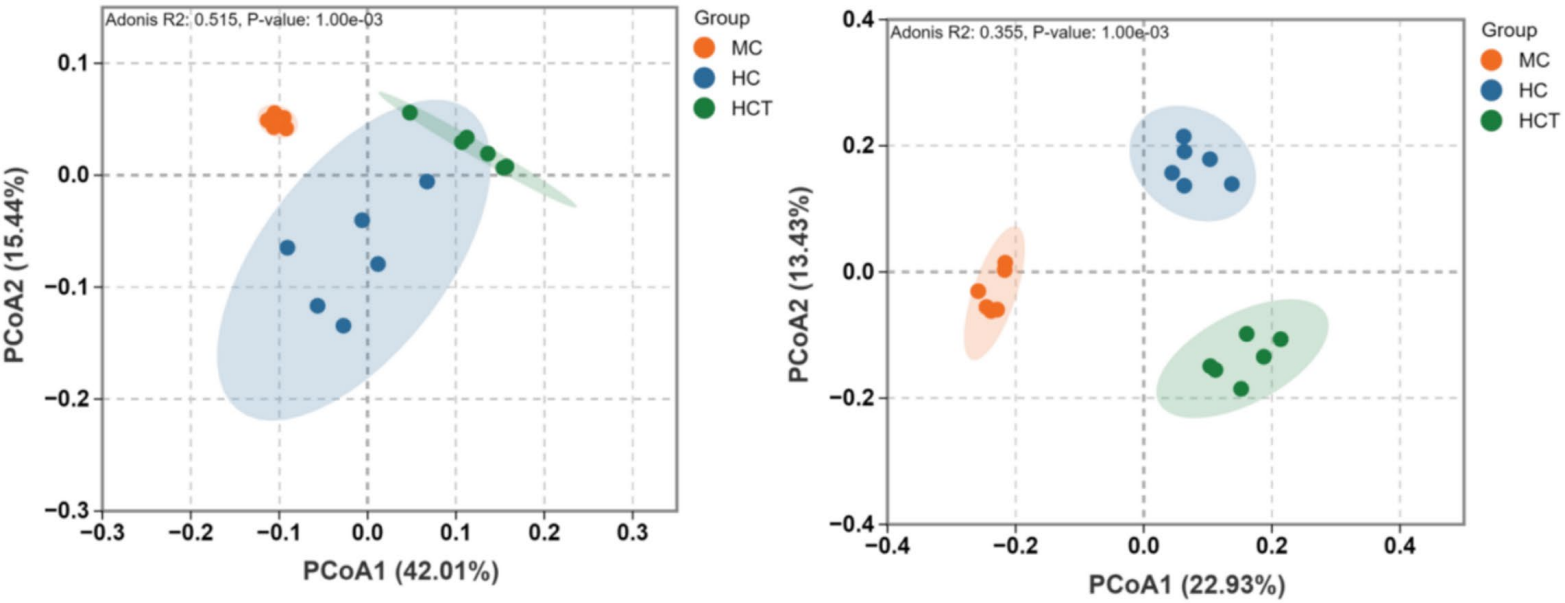
Effects of TASA on the *β*-diversity of the lamb colon flora. MC = (concentrate: roughage 50:50) diet; HC = (concentrate: roughage 70:30) diet; HCT = HC supplemented with 0.121 g/kg TASA.

According to the sequencing results of 16S rRNA, 1,441,812 raw reads and 898,168 high-quality sequences were obtained from the colonic contents of the three lamb groups, and 3,796 ASVs were generated after quality control. The sample species accumulation and rank abundance curves showed that the sampling was reasonable and sufficient (Supplementary Figure S1). To assess the effect of HCT on colonic microbial diversity, the sequences were analyzed for alpha diversity and beta diversity. As shown in Table 3, the Simpson, Chao, and Shannon indices in the HC group and HCT group were significantly lower than those in the MC group (*p* < 0.05), but there was no significant difference between the HC group and HCT group (*p* > 0.05). PCoA showed obvious separation of microbial communities in the three groups (*p* < 0.05, Figure 4). These findings indicate that high-concentrate diets and the addition of TASA to HC diets significantly affect the composition of the colonic microbiota in lambs.

**Table 3:** Alpha diversity index table of colonic bacteria.

Items	Groups			SEM	*P*-value
	MC	HC	HCT		
Simpson	1.00^a^	0.99^b^	0.99^b^	0.00	0.015
Chao	777.90^a^	604.04^b^	595.39^b^	28.54	0.007
Coverage (%)	1.00	1.00	1.00	0.00	0.221
Shannon	8.71^a^	8.05^b^	7.89^b^	0.11	0.002

Data were expressed as mean ± SEM. In the same row, values with different letter superscripts mean a significant difference (*p* < 0.05). MC = (concentrate: roughage 50:50) diet; HC = (concentrate: roughage 70:30) diet; HCT = HC supplemented with 0.121 g/kg TASA.

### 3.6 Effect of HCT on the relative abundance of bacterial communities in the colons of lambs

**Figure 5:**
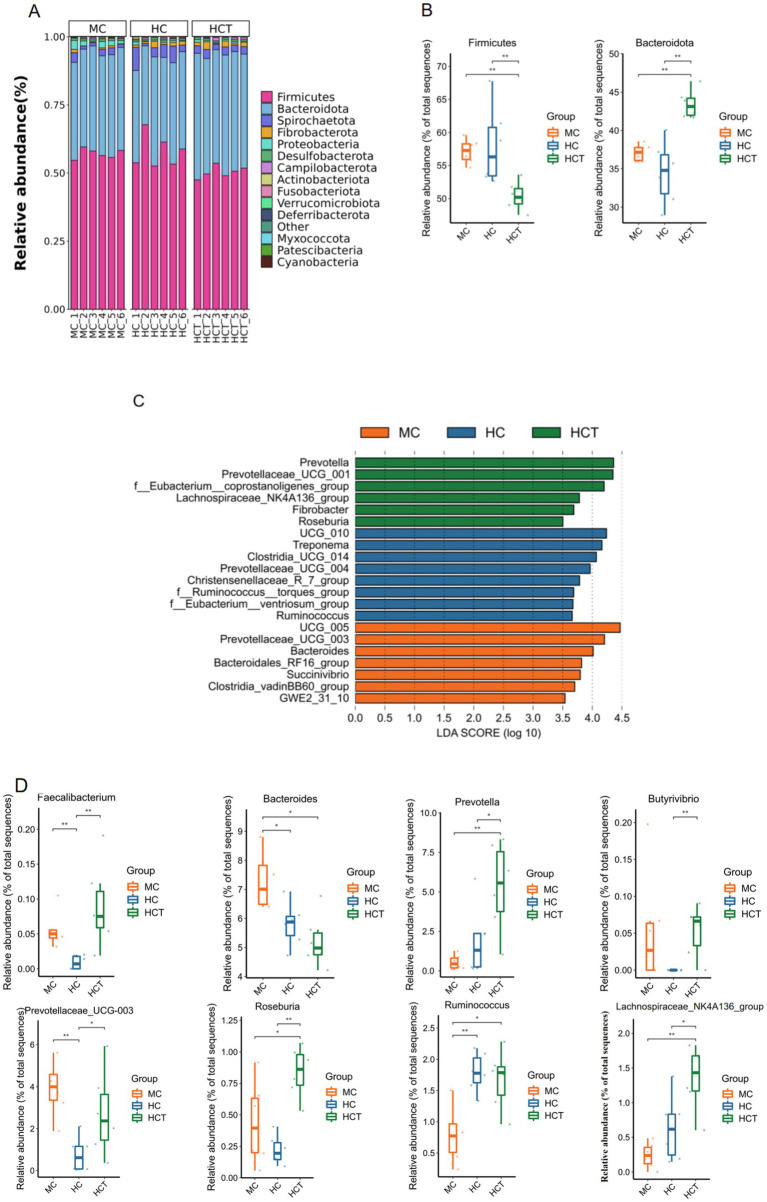
The addition of TASA altered the microbial composition of the lambs’ colons. **(A)** Colonic bacterial abundance distribution bar graph at the phylum level. **(B)** The Kruskal–Wallis test was used to assess the relative abundance of Firmicutes and Bacteroidota in the colonic contents of the lambs. **(C)** Bar graph of the LDA value distribution. **(D)** The Kruskal–Wallis test was used to determine the relative abundance of the dominant bacterial genera in the colons of the lambs. * *p* < 0.05, ** *p* < 0.01. MC = (concentrate: roughage 50:50) diet; HC = (concentrate: roughage 70:30) diet; HCT = HC supplemented with 0.121 g/kg TASA.

At the phylum level, the lamb colons were predominantly composed of Firmicutes, Bacteroidota, Spirochaetota, Proteobacteria, and Fibrobacterota (Figure 5A). The abundance of colonic Bacteroidota was significantly greater, whereas the abundance of Firmicutes was significantly lower in the HCT group (*p* < 0.01, Figure 5B). A total of 180 genera were identified at the genus level. The colonic microflora with notable discrepancies in each group of lambs were filtered out using LDA with a threshold of 3.5. *Prevotella*, *Prevotellaceae_UCG_001*, *Eubacterium__coprostanoligenes_group*, *Lachnospiraceae_NK4A136_group*, *Fibrobacter,* and *Roseburia* were enriched in the HCT group. *UCG_010*, *Treponema*, *Clostridia_UCG_014*, *Prevotellaceae_UCG_004*, *Christensenellaceae_R_7_group*, *Ruminococcus__torques_group*, *Eubacterium__ventriosum_group*, and *Ruminococcus* were enriched in the HC group. *UCG_005*, *Prevotellaceae_UCG_003*, *Bacteroides*, *Bacteroidales_RF16_group*, *Succinivibrio*, and *Clostridia_vadinBB60_group* were abundant in the MC group (Figure 5C). Significance tests were performed on the dominant bacterial genera in the colonic samples. The results are shown in Figure 5D; compared to the MC and HC groups, the abundance of *Faecalibacterium* and *Prevotellaceae_UCG-003* in the MC and HCT groups was significantly higher (*p* < 0.05 or *p* < 0.01). The abundance of *Prevotella*, *Roseburia*, and *Lachnospiraceae_NK4A136_group* in the HCT group was significantly higher (*p* < 0.05 or *p* < 0.01), and the abundance of *Butyrivibrio* was notably higher than in the HC group (*p* < 0.01). Compared to the MC group, the abundance of Bacteroidetes was significantly lower, while *the* abundance of *Ruminococcus* was significantly higher in the HC and HCT groups (*p* < 0.05 or *p* < 0.01).

### 3.7 Correlation analysis

**Figure 6:**
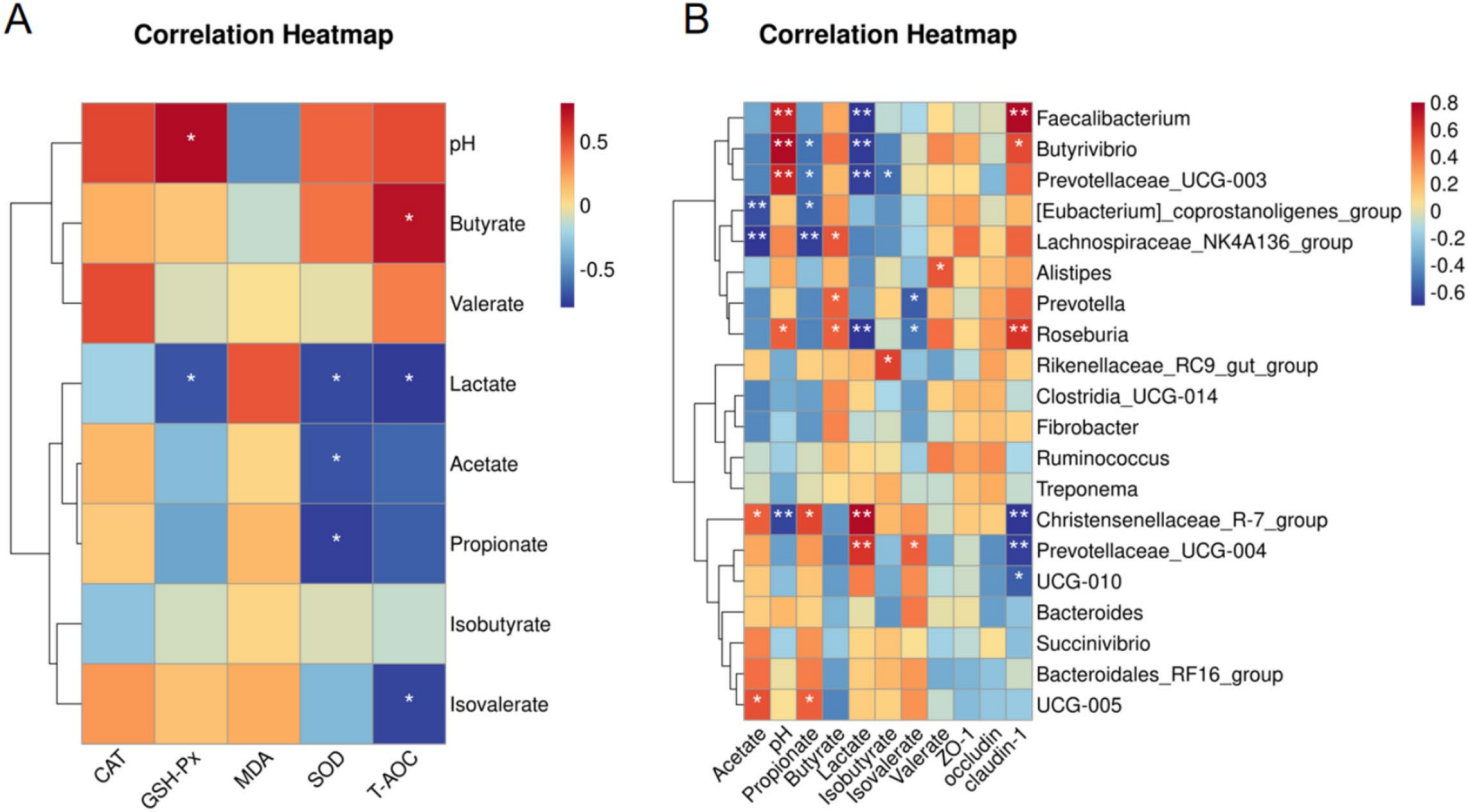
Correlation analysis. **(A)** Spearman correlation analysis was used to assess the correlation between colonic SCFA levels, pH, and antioxidant-related factors. **(B)** Spearman correlation between colonic SCFAs, tight junction proteins, and dominant genera. * *p* < 0.05, ** *p* < 0.01. MC = (concentrate: roughage 50:50) diet; HC = (concentrate: roughage 70:30) diet; HCT = HC supplemented with 0.121 g/kg TASA.

Spearman correlation analysis was used to examine how fermentation factors are related to antioxidant factors and the main types of bacteria. As shown in Figure 6, lactate was negatively correlated with SOD, T-AOC, and GSH-PX. pH was positively correlated with GSH-PX. T-AOC was positively correlated with butyrate and negatively correlated with isovalerate. There was a negative correlation between SOD and both acetate and propionate. *Prevotella*, *Roseburia*, and *Lachnospiraceae_NK4A136_group* were positively correlated with *butyrate*. *Roseburia*, *Faecalibacterium*, *Butyrivibrio*, and *Prevotellaceae_UCG-003* were strongly associated with higher pH values and lower lactate levels. C*hristensenellaceae_R-7_group* was negatively correlated with both lactate and pH but positively correlated with acetate and propionate. *[Eubacterium]_coprostanoligenes_group* and *Lachnospiraceae_NK4A136_group* were negatively correlated with acetate and propionate. In addition, claudin-1 was positively correlated with *Faecalibacterium*, *Butyrivibrio*, and *Roseburia* and negatively correlated with C*hristensenellaceae_R-7_group, Prevotellaceae_UCG-014,* and *UCG-010*.

## 4 Discussion

The colon is made up of a single layer of columnar epithelium, which makes it more vulnerable to the effects of HC diets. Previous studies have reported that feeding goats an HC diet causes detachment and damage to the colonic epithelium, widening of tight junction gaps, and swelling of mitochondria (17, 18). This experiment revealed that there were cavities and inflammatory cell infiltration in the colonic epithelium in the HC group, while the addition of TASA to the HC diet alleviated this phenomenon. Tight junctions are important components of the mucosal barrier between adjacent epithelial cells in the gastrointestinal tract and play a key role in regulating epithelial barrier permeability and preventing the translocation of LPS (19). HC diets can influence tight junction protein expression in the colon (3, 17). Claudins are key components of tight junctions, and they regulate the permeability and selective permeability of substances by maintaining tight connections between cells. When claudins decrease, the integrity of tight junction structures and functions is disrupted, leading to a decline in barrier function (20). This study found that claudin-1 protein expression in the colonic epithelium was significantly higher in the HCT group than in the MC and HC groups, suggesting that TASA may enhance colonic epithelial barrier function by regulating the expression of claudins in the colonic epithelium.

HC diets interfere with the redox balance in the colon, causing oxidative damage to the colonic epithelium. The main manifestations are decreased intestinal antioxidant enzyme activity, increased lipid peroxidation products, and changes in metabolic pathways that affect antioxidant capacity (6). HC diets lead to the production of large amounts of LPS and biogenic amines in the gastrointestinal tract (21). These substances increase the accumulation of ROS by activating NADPH oxidase and xanthine oxidase or directly destroying mitochondrial function (22, 23). In addition, proinflammatory factors activated by LPS can exacerbate oxidative stress by increasing the expression of ROS-generating enzymes (24). In goats fed HC diets, the levels of LPS and MDA in the hindgut epithelium are reportedly increased, whereas the activities of GSH-Px and SOD are reduced (25). *Sophora alopecuroides* alkaloids exert antioxidant effects by scavenging hydroxyl radicals and enhancing antioxidant enzyme activity. *Sophora alopecuroides* alkaloids have been shown to reduce the production of ROS and MDA in the primary cultured hippocampal neurons of neonatal rats while increasing the activity of CAT, SOD, GSH-Px, and T-AOC, thereby providing neuroprotective effects (26). Cui et al. (27) reported that *Sophora alopecuroides* alkaloids inhibited the production of ROS in LPS-treated mouse alveolar epithelial cells. An et al. (16) reported that HC diets reduced liver SOD activity, whereas the addition of SA to the HC diet increased liver GSH-Px and T-AOC activities. In this study, the HC diet led to an increase in the MDA concentration and a decrease in antioxidant enzyme activity in the colon. However, adding TASA to the HC diet decreased the MDA concentration and increased antioxidant enzyme activity. These results suggest that prolonged feeding of HC diets leads to increased levels of lipid peroxidation products and decreased antioxidant enzyme activity. TASA can improve the antioxidant capacity of the colonic epithelium and reduce oxidative damage.

Moreover, the gut microbiota, as a key regulator of host metabolism and immunity, is closely linked to intestinal health. Therefore, we further studied the effect of adding TASA to an HC diet on the colonic microbiota of lambs. The colon is a key site for water and electrolyte absorption, participates in fecal formation, and maintains fluid balance. It also breaks down complex carbohydrates into SCFAs. Multiple studies have shown that after ruminants consume high-concentrate diets, lactic acid and VFA concentrations in the colon increase, while the pH level decreases (28, 29). We observed that supplementation with TASA in HC diets increased colonic pH and butyrate concentrations while decreasing acetate, propionate, and lactate concentrations. Acetate and propionate are the major VFAs involved in colonic fermentation. A decrease in pH is usually associated with increased lactic acid and VFA levels (2), which explains the increase in pH in the HCT group. The reduction in acetate, propionate, and lactate concentrations in the HCT group may be due to the antimicrobial effects of SA alkaloids. However, beta diversity analysis revealed that adding TASA to HC diets could regulate the structure and abundance of the colonic microflora in lambs. Butyrate promotes the repair and regeneration of epithelial cells (30), has anti-inflammatory effects, and enhances gut barrier function (31, 32). This study revealed that the elevated butyrate concentration in the HCT group implies that TASA are actively involved in maintaining lamb colon health. Firmicutes and Bacteroidetes are the dominant bacterial phyla present in ruminant colons (3). Bacteroidetes encode many CAZymes that specialize in the degradation of complex polysaccharides, whereas Firmicutes secrete various glycoside hydrolases (HHs) and cellulases that specialize in cellulose degradation (33, 34). We found that in the HCT group, the abundance of Bacteroides increased significantly, whereas that of Firmicutes decreased, which may help alleviate the accumulation of organic acids in the hindgut (33). A change in the ratio of Firmicutes to Bacteroidetes can inhibit the growth of harmful bacteria, helping to maintain the dynamic balance of the microbiota (35). A previous study showed that in the probiotic-supplemented group, the abundance of Bacteroidetes in the rumen of Sunite sheep increased, whereas the abundance of Firmicutes decreased (36). In our previous study, supplementing an HC diet with SA increased Bacteroides and decreased Firmicutes abundance in the lamb rumen (13), which is consistent with the present findings.

LEfSe analysis and genus-level analysis revealed that *Treponema* was significantly enriched in the HC group. *Treponema* includes several pathogenic species, and the increased abundance due to HC diets may negatively affect intestinal health (37). *Prevotellaceae_UCG-003* has been identified as a key genus distinguishing SARA-susceptible goats from healthy goats, with the abundance of *Prevotellaceae_UCG-003* in the rumen of SARA goats being significantly lower than that in healthy goats (38). *Prevotella* species can ferment carbohydrates and participate in the synthesis of amino acids and lipids (38). Higher levels of *Prevotella* can activate dendritic cells through the production of succinate, thereby modulating intestinal inflammation (39). In this study, we discovered that the abundance of *Prevotellaceae_UCG-003* was much lower in the HC group, but when TASA was added to the HC diet, the abundance of *Prevotellaceae_UCG-003* and *Prevotella* in the colon increased significantly. *Faecalibacterium* is a probiotic, and its main fermentation product is butyrate. In addition, acetate consumption is a major driving force through which *Faecalibacterium* members produce butyrate in the healthy human gut (40, 41). Butyrate is the main energy source for the colonic epithelium and plays a role in maintaining the epithelial barrier, regulating the immune system, and reducing inflammation (42, 43). In addition, butyrate can activate the intracellular Nrf2 signaling pathway, initiate the expression of the downstream endogenous antioxidant enzyme, and counteract oxidative stress (44). *Faecalibacterium* can secrete microbial anti-infective molecules, which can directly inhibit the NF-κB signaling pathway, thereby playing a significant anti-inflammatory role (45). Inflammation and oxidative stress often promote each other, forming a “vicious cycle.” In the process of inflammation, immune cells produce a lot of reactive ROS, and excessive ROS further aggravates tissue inflammation. By inhibiting the NF-κB pathway, fecal *Faecalibacterium* can significantly reduce the production of ROS, thereby decreasing oxidative stress and inflammatory reaction. Numerous studies have linked reduced *Faecalibacterium* abundance to inflammatory bowel disease (IBD) (46, 47). Furthermore, evidence indicates that *Faecalibacterium* induces the differentiation of dendritic cells into IL-10-producing Treg and Tr1 cells and suppresses proinflammatory cytokine production to alleviate IBD (48). In this study, the abundance of *Faecalibacterium* in the MC and HCT groups increased. Furthermore, the addition of TASA to the HC diet increased the abundance of *Roseburia*, *Lachnospiraceae_NK4A136_group*, and *Butyrivibrio* in the colonic contents. *Roseburia* is a key producer of butyrate in the gut, and it contributes to immune regulation and exerts anti-inflammatory effects through butyrate production (49). In a DSS-induced colitis mouse model, oral administration of *Roseburia*-derived extracellular vesicles improved colitis by downregulating NF-κB and STAT3 in colonic tissues (50). *Lachnospiraceae_NK4A136_group* and *Butyrivibrio* are also key butyrate-producing bacteria in the gut microbiome. *Lachnospira_NK4A136_group* expression is positively correlated with tight junction protein and anti-inflammatory factor expression and negatively correlated with proinflammatory cytokines, suggesting that increasing the expression of *Lachnospiraceae_NK4A136_group* helps reduce intestinal permeability and inhibits intestinal inflammation (51, 52). In summary, the addition of TASA to HC diets may alleviate colonic damage caused by HC diets by increasing the abundance of butyrate-producing bacteria in the colonic microflora.

## 5 Conclusion

In conclusion, supplementing HC diets with TASA enhances the colon barrier and antioxidant function, reduces the inflammatory response, and improves the intestinal flora structure by increasing the abundance of butyrate-producing bacteria, thereby alleviating colonic damage caused by HC diets.

## Data Availability

The datasets presented in this study are available in online repositories, the names of the repository/repositories and accession number(s) can be found at: PRJNA1188773.
