# Supplementary material for: The role of Sophora alopecuroides alkaloids in colon health of lambs fed high-concentrate diets for extended periods: impact on barrier function, antioxidation, and microflora
Source: Front Vet Sci. 2025 Dec 10;12:1698892. doi: 10.3389/fvets.2025.1698892 (PMC12729053; doi:10.3389/fvets.2025.1698892)
Supplement: Supplementary file 1 [file Image_1.pdf]

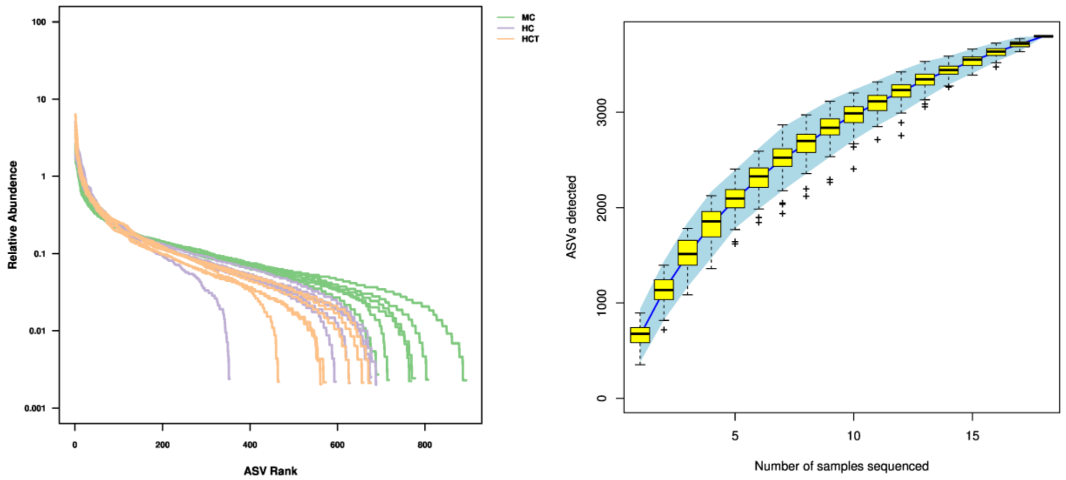

Figure S1 Relative bacterial richness and evenness analyses. (A) Rank abundance curve. (B) Species accumulation curve.
